# Complex Cytokine Responses in Imported Scrub Typhus Cases, Germany, 2010–2018

**DOI:** 10.4269/ajtmh.19-0498

**Published:** 2019-11-25

**Authors:** Philip Eisermann, Jessica Rauch, Stefan Reuter, Lukas Eberwein, Ute Mehlhoop, Petra Allartz, Birgit Muntau, Dennis Tappe

**Affiliations:** 1 National Reference Center for Tropical Pathogens, Bernhard Nocht Institute for Tropical Medicine, Hamburg, Germany;; 2 4th Department of Internal Medicine, Klinikum Leverkusen gGmbH, Leverkusen, Germany

## Abstract

Scrub typhus is a life-threatening zoonotic disease, which is caused by *Orientia tsutsugamushi*, an obligatory intracellular Gram-negative bacterium. It is transmitted by *Leptotrombidium* mites in endemic regions of Southeast Asia. So far, data on imported scrub typhus cases to non-endemic areas and immunological descriptions are rare. Eleven scrub typhus cases that had been diagnosed by the German National Reference Center for Tropical Pathogens between 2010 and 2018 were retrospectively reviewed for clinical symptoms, laboratory changes, and travel destinations. Patient sera were included if follow-up samples showed simultaneous seroconversion for IgM and IgG antibody responses by immunofluorescence assays or concurrence with the first serum sample. The median of seroconversion was week 2 after symptom onset. Cytokine levels were measured over time, demonstrating simultaneously upregulated major Th1, Th2, and Th17 cytokines in the acute phase of infection followed by normalization during convalescence. This study underlines the complex mixed cytokine response elicited by scrub typhus and highlights clinical and diagnostic aspects of imported infections with *O. tsutsugamushi*.

## INTRODUCTION

Scrub typhus is a vector-borne infection caused by *Orientia tsutsugamushi*. The obligate intracellular bacterium is transmitted by bites of larval stage *Leptotrombidium* mites. The inoculation of the bacteria causes a local inflammatory reaction often resulting in a necrotic lesion, the eschar. Systemic manifestations occur 5–14 days after infection, comprising most frequently fever, a maculopapular rash, lymphadenopathy, headache, and myalgia. Severe complications such as myocarditis, acute renal failure, pneumonia, and meningoencephalitis might develop, which can lead to multiple organ failure with a high case fatality rate if not adequately treated. Scrub typhus is endemic in a triangular geographic region between eastern Russia, Japan, Pakistan, and Australia.^1^ However, confirmed autochthonous *Orientia* sp. infections have been recently described in Chile^2^ and the United Arab Emirates.^3^ Furthermore, there are also reports about possible scrub typhus cases in East Africa,^4,5^ underlining the emerging nature of this disease. Little is known about the immunology of human infection with *O. tsutsugamushi*, however.

In this study, we retrospectively analyzed imported scrub typhus cases that had been diagnosed at the National Reference Center for Tropical Pathogens between 2012 and 2018. Clinical data were collected, and cases were serologically analyzed. Antibody kinetics was determined from follow-up sera. Serum cytokine responses were measured by flow cytometry from all available sera.

## MATERIALS AND METHODS

### Ethics statement.

All data analyzed were anonymized. Informed consent was obtained from all individual participants included in the study.

### Cases and inclusion criteria.

The database of the German Reference Center for Tropical Pathogens at the Bernhard Nocht Institute for Tropical Medicine, Hamburg, was screened for imported scrub typhus cases diagnosed from January 1, 2010 to December 31, 2018. Scrub typhus cases were defined as a clinically compatible disease with at least one of the following laboratory test results: a positive polymerase chain reaction (PCR) testing, a seroconversion to *O. tsutsugamushi* antigens in an indirect immunofluorescence antibody test (IFAT), a parallel IgM and IgG detection against *O. tsutsugamushi* antigens in a single sample using IFAT, or a single IFAT IgG or total Ig titer of ≥ 1:320. In addition, serology for typhus group and spotted fever group rickettsiae (in-house IFATs), leptospirosis (in-house ELISA), and dengue fever (in-house IFAT) had to be negative.

### Serological and molecular assays.

In-house scrub typhus IFAT was performed using *O. tsutsugamushi* strain Karp in L929 mouse fibroblast cell culture. Immunofluorescence antibody test reference values were < 1:40 (IgM) and < 1:80 (IgG and total Ig). All the reference values were determined with sera from 200 healthy Caucasian blood donors. *Orientia tsutsugamushi*–specific real-time quantitative PCR (qPCR) was performed targeting the 56 kDa antigen gene.^6^ Depending on the clinical case and the available sample material, qPCRs were performed with DNA extracts from EDTA blood, cerebrospinal fluid, or eschar biopsy samples.

### Cytokine measurements.

Serum cytokine responses were analyzed by LEGENDplex (BioLegend, Fell, Germany) from all available sera. Thirteen sera from healthy blood donors served as controls. For cytokine analysis, blood sampling dates of the patients were assigned to the acute phase of infection (weeks 1–4 after symptom onset) and convalescent phase (> 4 weeks after symptom onset). The standard detection limits of the LEGENDplex assay for the analyzed cytokines were as follows: basic fibroblast growth factor (24.2 pg/mL), eotaxin (6.9 pg/mL), granulocyte colony-stimulating factor (G-CSF, 10.7 pg/mL), granulocyte–macrophage colony-stimulating factor (GM-CSF, 6.7 pg/mL), interferon-α (IFNα, 1.2 pg/mL), interferon-γ (IFNγ, 5.2 pg/mL), interleukin (IL) 1ß (4.1 pg/mL), IL-2 (4.3 pg/mL), IL-4 (4.8 pg/mL), IL-5 (4.0 pg/mL), IL-6 (4.4 pg/mL), IL-8 (1.5 pg/mL), IL-9 (4.0 pg/mL), IL-10 (3.9 pg/mL), IL-12p70 (19.5 pg/mL), IL-13 (3.3 pg/mL), IL-17A (3.7 pg/mL), IL-17F (5.0 pg/mL), IL-21 (6.4 pg/mL), IL-22 (19.5 pg/mL), IFNγ-induced protein 10 (IP-10, N/A), monocyte chemotactic protein-1 (MCP-1, N/A), macrophage inflammatory protein-1α (MIP-1α, 5.0 pg/mL), macrophage inflammatory protein-1β (MIP-1β, 5.2 pg/mL), platelet-derived growth factor BB (PDGF-BB, N/A), regulated on activation, normal T cell expressed and secreted (RANTES, 5.2 pg/mL), tumor necrosis factor-α (TNFα, 4.4 pg/mL), and vascular endothelial growth factor (VEGF, 26.9 pg/mL).

Statistical analysis was performed with GraphPad Prism 7 (GraphPad Software Inc., La Jolla, CA). For comparison between the analyzed groups, the Kruskal–Wallis test and subsequent Dunn’s multiple comparisons test were used.

## RESULTS

A total of 11 scrub typhus patients were identified (Table 1), with an age range of 19–70 years (mean age 42.5 years) and a male : female ratio of 2.7:1. Scrub typhus was acquired during travel in Southeast Asia (Thailand, Laos, Vietnam, South Korea, Malaysia, and Myanmar) and Nepal. The exposure to mites had not been specifically asked.

**Table 1:** Characteristics of 11 patients with imported scrub typhus, Germany, 2010–2018*

Patient no.	Age, year/gender	Year of diagnosis	Travel history	PCR result†	Signs and symptoms	Hospitalized
1	55/M	2018	Laos	Positive‡	Eschar, exanthema, fever, and headache	Yes
2	50/F	2018	Laos and Thailand	ND	Exanthema, fever, headache, and splenomegaly	Yes
3	34/M	2017	Vietnam	ND	Exanthema, fever, and diarrhea	Yes
4	35/M	2016	Thailand and Malaysia	ND	Exanthema, fever, severe headache, lymphadenopathy, myalgia, and arthralgia	No
5	70/M	2015	Nepal	ND	Fever, headache, lymphadenopathy, arthralgia, and splenomegaly; sequelae on last follow-up examination	Yes
6	25/M	2014	South Korea	ND	Eschar and fever	No
7	27/F	2014	Thailand	ND	Fever and severe headache	Yes
8	51/M	2014	Thailand	Negative§	Ataxia, bilateral facial paresis, diplopia, ophthalmoplegia, tetraparesis, and respiratory failure; sequelae on last follow-up examination	Yes
9	61/M	2014	Myanmar	Positive¶	Eschar, exanthema, fever, and arthralgia	Yes
10	19/M	2013	Vietnam	ND	Eschar, exanthema, and fever	No
11	41/F	2012	South Korea	ND	Eschar and fever	Yes

PCR = polymerase chain reaction; M = male; F = female.

* ND = not performed (whole blood or biopsy sample not available).

† Quantitative PCR targeting the 56 kDa gene of *Orientia tsutsugamushi*.

‡ Positive from eschar biopsy sample.

§ Negative from cerebrospinal fluid.

¶ Positive from eschar biopsy sample and whole blood.

**Figure 1.**
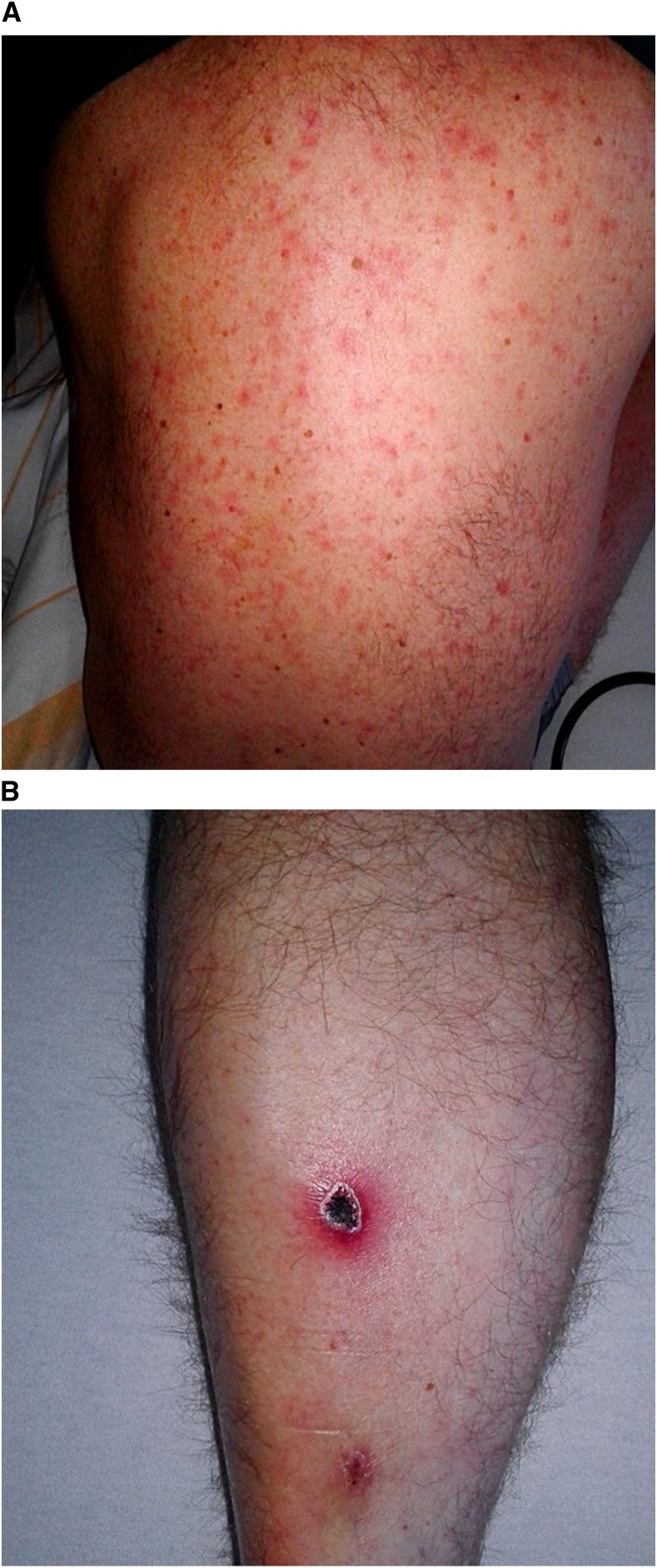
Typical exanthema and eschar in a scrub typhus patient (patient 1). (**A**) Maculopapular and non-pruritic rash on the back. (**B**) Eschar located on the shin of the same patient after travel to Laos.

Most patients had fever on admission (10 patients, 91%) followed by exanthema (6 patients, 55%; Figure 1A), headache (5 patients, 45%), eschar (5 patients, 45%; Figure 1B), and myalgia/arthralgia (3 patients, 27%). The most common combination of symptoms was fever and headache, which was seen in five patients (45%). Lymphadenopathy and splenomegaly were found in two patients (18%). Neurological signs, respiratory failure, and diarrhea were noted in one patient each. Hospitalization was necessary in a total of eight patients (73%). Patients had received doxycycline treatment (200 mg/d for 5–21 days) in the country of travel or on return. After treatment, patient 5 showed arthralgia and mild cephalgia, whereas patient 8 presented with an incomplete tetraparesis at the final clinical follow-up examination. All other patients recovered from the infection without sequelae.

Laboratory changes were found in the medical records of six patients on admission and included increased levels of C-reactive protein (CRP) and elevated liver enzymes in five patients (83%). Anemia, increased concentrations of lactate dehydrogenase (LDH), and thrombocytopenia were seen in two patients (33%), whereas eosinopenia was present in one patient (17%). Of note, patients were observed at different days of illness in different hospitals.

Polymerase chain reaction was performed on clinical samples from three patients. A positive PCR result for *O. tsutsugamushi* was obtained in two cases, from an eschar biopsy and whole blood in patient 9 and from an eschar biopsy sample alone in patient 1 (Table 1). No genotyping of *O. tsutsugamushi* was performed.

Scrub typhus serology was performed in nine patients (82%). From the two PCR-positive individuals, no serum was available. Antibodies against *O. tsutsugamushi* antigens were detected earliest in week 1 of illness, and the median of seroconversion was week 2. Seroconversion (with parallel IgM and IgG detection) was observed in three patients (33%), whereas the presence of IgM and IgG in the first sample was seen in five patients (56%). In one patient (patient 8), only IgM and total Ig were positive, with no specific IgG seroconversion after four weeks of illness.

**Figure 2.**
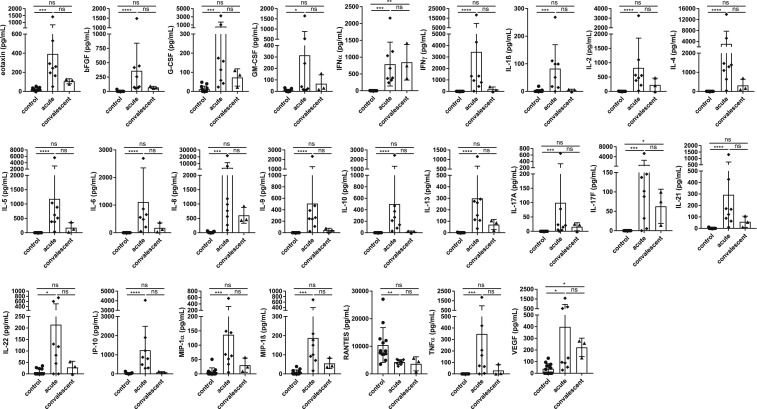
Cytokine and chemokine levels in imported scrub typhus cases. Eleven sera from nine patients with scrub typhus and 13 sera from healthy blood donors (controls) were analyzed in parallel by bead-based LEGENDplex assay. Eight serum samples were assigned to the acute phase of scrub typhus (weeks 1–4 after symptom onset) and three samples to the convalescent phase of the infection (> 4 weeks after symptom onset). Most serum cytokine and chemokine levels started to increase during the acute phase of illness and decreased again in the convalescent phase. Data are expressed as mean ± SD. Statistical analyses were performed with the Kruskal–Wallis test and subsequent Dunn’s multiple comparisons test. Asterisks indicate statistically significant differences (*P < 0.05, **P < 0.01, ***P < 0.001, ****P < 0.0001); ns = not significant.

Serum cytokines could be measured in nine patients, in two of them also at two different time points. Eight serum samples were assigned to the acute phase of illness and three samples to the convalescent phase of the infection. Almost all the measured serum cytokines and chemokines were significantly elevated in patients in the acute phase of illness in comparison with healthy controls (Figure 2). These include the serum levels of eotaxin, FGFb, G-CSF, GM CSF, IFNα, IFNγ, IL-1ß, IL-2, IL-4, IL-5, IL-6, IL-8, IL-9, IL-10, IL-13, IL-17A, IL-17F, IL-21, IL-22, IP-10, MIP-1α, MIP-1ß, TNFα, and VEGF. Although the concentrations of IFNα, IL-17F, and VEGF remained elevated in the convalescent phase of the infection, levels of all other chemokines/cytokines decreased again and were comparable with the concentrations of the control group (Figure 2). The levels of IL-12, PDGF BB, and MCP-1 were similar in both the acute and convalescent phases in patients and controls (data not shown). Of note, the IL-12 concentrations were only elevated in the serum of two patients in the acute phase of illness (patient 1, 226.7 pg/mL and patient 4, 130.4 pg/mL). Interestingly, the levels of RANTES were found to be significantly reduced in the acute phase of illness compared with healthy controls. In the serum of one patient (patient 5), the concentrations of most cytokines and chemokines were markedly higher during the acute phase of infection than in the other patients.

## DISCUSSION

Reports about imported scrub typhus cases to non-endemic regions are rare. According to a recent review of the available data, fewer than 40 patients have been reported.^7^ The overall majority of published data describe imported infections from the traditional area of the tsutsugamushi triangle.^7–11^ In line with these reports, all infections with *O. tsutsugamushi* in our study were acquired in countries located in Southeast Asia and in Nepal. In contrast to the few reported cases in travelers, at least one million cases of scrub typhus occur in the Asian–Pacific region annually.^12^ Insufficient clinical experience and unavailable diagnostic methods in many non-endemic regions might explain this lack of published data. Unarguably, scrub typhus is an important differential diagnosis in travelers returning from endemic regions presenting with an acute febrile illness. Furthermore, a report about an imported *Orientia* sp. infection from the Middle East to Australia^3^ and autochthonous scrub typhus cases in Chile^2^ should raise awareness of the emergence of this disease in unexpected regions.

Most patients in our study presented with nonspecific symptoms such as fever (91%), exanthema (55%), and headache (45%). Prevalence of the characteristic eschar in scrub typhus patients can vary greatly^13–15^ and might depend on the *O. tsutsugamushi* genotype.^16^ In our study, nearly half of the patients presented with an eschar. In patient 8, an eschar was not detected and treatment with doxycycline was delayed. This patient developed meningoencephalitis and an acute respiratory distress syndrome, leading to prolonged recovery with incomplete tetraparesis. The absence of an eschar seems to be associated with more severe symptoms and complications by hindering swift diagnosis and early commencement of adequate treatment.^17,18^ Meningoencephalitis is a well-documented complication of scrub typhus. The clinical outcome is generally benign, but chronic sequelae and fatalities might occur.^19^

Laboratory data were not available from all patients. Increased levels of CRP and liver enzymes were detected in more than 80% of patients, followed by anemia, thrombocytopenia, and elevated serum concentration of LDH. These results are in line with previous reports about imported scrub typhus cases.^7,8^ According to studies in endemic areas, laboratory changes are not as frequent as in imported cases, but more studies with larger numbers of imported *O. tsutsugamushi* infections are needed to draw further conclusions.^20,21^

Detection of antibodies against *O. tsutsugamushi* by IFAT is the gold standard in diagnosing scrub typhus.^22^ IgM seroconversion is usually detectable by the end of the first week after symptom onset, followed by IgG near the end of the second week.^23^ In our study, the earliest detection of antibodies was in the first week and the median time of seroconversion was week 2. Of importance, serum from each patient had not been collected on the same day, so the data can only serve as estimates. In more than half of the individuals, IgG and IgM were already present in the first serum sample. In the remaining patients, IgM and IgG seroconverted simultaneously, except for patient 8 who did not show IgG seroconversion and who suffered from scrub typhus–associated meningoencephalitis. The reason for the lack of IgG seroconversion and whether this was a predisposition for the severe clinical course remain unsolved. Because of the delayed antibody response, clinical diagnosis can often only be confirmed in retrospect.^24^ Direct early pathogen detection before seroconversion by PCR might initiate immediate effective treatment. Here, eschar biopsy samples from two patients were analyzed by PCR, with positive results. Samples from the remaining three individuals, who presented with an eschar, were not available. Several studies have shown that PCR using eschar tissue is more sensitive than PCR with blood samples.^23^ We were also able to detect *O. tsutsugamushi* DNA in a whole blood sample during the acute phase of the disease before antibiotic treatment was started. However, after treatment is initiated, pathogen detection by PCR in blood samples usually is not possible, whereas eschar tissue PCR still can yield positive results for up to 7 days, enlarging the diagnostic window.^25^

Here, we report data on the systematic inflammatory response in scrub typhus patients in the acute and convalescent phases of illness. To our knowledge, this is the first study to measure a broad spectrum of cytokines and chemokines in a case series of imported scrub typhus. The investigation shows that 22 human cytokines and chemokines, including the major Th1, Th2 and Th17 cytokines, were simultaneously upregulated in the sera of patients in the acute phase and declined again after 4 weeks during convalescence. Why most cytokines and chemokines were markedly elevated in the serum from the acute phase of the elderly patient 5 is unclear. This patient had anemia, thrombocytopenia, and eosinopenia and showed elevated liver enzymes and increased CRP and LDH concentrations. He suffered later from relatively mild sequelae.

Significantly increased serum levels of IFNγ and TNFα in the acute phase in combination with elevated IL-12 production, at least in two patients, are consistent with a Th1 immune pattern. The expression of IFNγ seems to be protective against *O. tsutsugamushi* infection in mice,^26–28^ and our findings confirm previously published data that the IFNγ concentration is elevated in the serum of scrub typhus patients during the acute phase.^6,29^ The role of TNFα is not as clear during the *O. tsutsugamushi* infection: On the one hand, TNFα can propagate a strong Th1 response, leading to elimination of the pathogen, but on the other hand, serum concentrations of TNFα correlate with the severity of scrub typhus, indicating a fatal role of TNFα in the pathogenesis.^29^ In addition, increased levels of cytokines such as IL-1β, IL-4, IL-6, IL-13, IL-17, IL-21, and IL-22 in the sera of our patients underline a simultaneous Th2 and Th17 response, so Th1-, Th2-, and Th17-type responses do not seem to be clearly polarized. IL-1ß, IL-6, and IL-21 have been shown to initiate the differentiation of T cells toward the Th17 lineage, and the main effector cytokines of Th17 cells are IL-17 and IL-22.^30–32^ Th17 cells became the focus of attention as they play a protective role during bacterial infections; however, they seem to mediate tissue damage.^33–36^ A correlation of pathologies such as hepatitis with high IL-17 levels in the serum of *O. tsutsugamushi*–infected patients could not be found by others,^37^ so it is likely that the Th17-type response is rather beneficial for the host than detrimental. In addition, a number of chemokines were found to be upregulated during the acute phase of infection, including IL-8, IP-10, MCP-1, MIP-1α, and MIP-1β. These chemokines attract leukocytes such as neutrophils and macrophages into the inflamed tissue and promote leukocyte–endothelial cell interaction during inflammation. Interestingly, MIP-1α, MIP-1β, and MCP-1 correlated with vascular inflammatory cell infiltration in in vivo studies in murine models of *O. tsutsugamushi* infection,^27^ and this could contribute to vascular permeability observed in patients with severe scrub typhus. By contrast, the expression of the chemokine RANTES was significantly reduced in the acute and convalescent phases in our patients, which has also been detected in scrub typhus patients before, associated with disease severity and fatal outcome.^38^

However, not only pro-inflammatory mechanisms were initiated during the acute phase of infection but also anti-inflammatory responses. Th2-derived cytokines such as IL-4, IL-5, IL-6, and IL-13 are not just essential for B cell differentiation and isotype switching, but IL-4 is also described to limit or attenuate tissue damage due to its anti-inflammatory properties, which include the suppression of pro-inflammatory and Th1-type responses.^26,39,40^ In addition, the observed high expression of the anti-inflammatory cytokine IL-10 in the acute phase of infection in our patients and also other studies addressing scrub typhus represents an additional counterbalancing mechanism to ensure homeostasis within the host.^6,29,38,41^ Its antagonistic effect against further pro-inflammatory cytokine production, also confirmed by the detection of the diminished cytokine production in the convalescent phase in our patients, exerts an inhibitory mechanism on the immune response and potentially prevents further pathological tissue alterations. Of note, these mechanisms can lead to diminished clearance of the pathogen, but they could also prevent overshooting immune reactions and, thus, reduce damage during clearance of the pathogen.

In conclusion, our study shows a mixed cytokine pattern in acute scrub typhus cases. More studies, including T cell response analyses, are needed to shed more light on the immunology and pathophysiology of scrub typhus. In addition, the presented clinical and diagnostic data may guide clinicians in non-endemic countries toward a swift diagnosis and the initiation of early effective treatment procedures.
